# Complete Genome Sequence of a Papio anubis Simian Foamy Provirus

**DOI:** 10.1128/MRA.01063-19

**Published:** 2019-10-03

**Authors:** Brice Jegado, Renaud Mahieux

**Affiliations:** a International Center for Research in Infectiology, Retroviral Oncogenesis Laboratory, INSERM U1111-Université Claude Bernard Lyon 1, CNRS, UMR5308, Ecole Normale Supérieure de Lyon, Université Lyon, Fondation pour la Recherche Médicale, LabEx Ecofect, Lyon, France; Portland State University

## Abstract

The full-length sequence of a Papio anubis simian foamy provirus was obtained by using PCR followed by Sanger sequencing. This simian foamy virus from a P. anubis animal (SFVp.anubis) is 13,393 bp long. Like other proviruses, the genome of SFVp.anubis is organized with long terminal repeats (LTRs), as well as *gag*, *pol*, *env*, *tas*, and *bet* genes. SFVp.anubis is closer to Old World African strains than to New World ones.

## ANNOUNCEMENT

Similar to humans, nonhuman primates (NHPs) can be infected with the following three different families of exogenous retroviruses: simian immunodeficiency virus (SIV), simian T cell leukemia virus (STLV), and simian foamy virus (SFV), the counterparts of human immunodeficiency virus (HIV), human T cell leukemia virus (HTLV), and human foamy virus (HFV), respectively. However, and contrary to HIV and HTLV-1, the *Spumavirus* HFV is in fact still a simian virus, given the fact that it always originates from current contacts with NHPs and cannot be transmitted from human to human. SFV infection is asymptomatic, although a recent report described lower levels of hemoglobin in infected humans (1). SFV seroprevalence can reach >75% in captive and wild NHPs (2), and at least 31 and 45 NHP species are infected with STLV-1 and SIV, respectively (3, 4). Since these three retroviruses have common modes of transmission, coinfections are frequent in NHPs and might have a biological impact (5). As an example, it was recently shown that STLV-1/SFV natural coinfection leads to an increased SFV proviral load (6). Only partial sequences of baboon SFV were previously reported (6–8). Full-length foamy virus sequences obtained here from naturally infected captive baboons (Papio anubis housed at the primate center of the Centre National de la Recherche Scientifique [CNRS] [UPS 846] in Rousset-sur-Arc, France) will be useful for performing further investigation on coinfection consequences.

**FIG 1:**
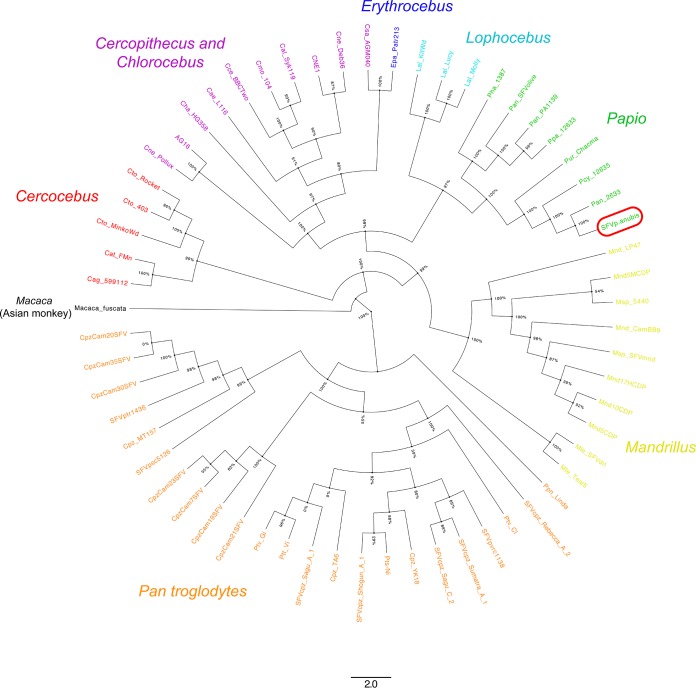
Phylogenetic relationships between 409 bp of SFV integrase sequences among Old World NHP species. All sequences were aligned by ClustalW with Seaview software (sequences from 61 NHPs infected by SFV). The phylogenetic tree was constructed using the maximum-likelihood method (PhyML) with percentage values and edited with FigTree v1.4.4. Values next to branches are bootstrap values. Each color represents a given NHP genus, as follows: Macaca (black), Cercocebus (red), Cercopithecus and Chlorocebus (pink), Erythrocebus (dark blue), Lophocebus (light blue), Papio (green), Mandrillus (yellow), and Pan troglodytes (orange). The sequence circled in red (for SFVp.anubis, a simian foamy virus from a P. anubis animal.) represents the integrase sequence from our complete SFV genome.

The use of animals was approved by the ethics committee (Autorisation de Projet Utilisant des Animaux à des Fins Scientifiques [APAFIS] number 4227-201604130940121) of the French Ministry of Higher Education, Research, and Innovation. The experimental procedure complied with current French laws and European directive 86/609/CEE. Animals were cared for in compliance with French regulations and were anesthetized with ketamine and medetomidine to allow blood draw. Peripheral blood mononuclear cells (PBMCs) were isolated by Ficoll gradient, washed, and lysed. Genomic DNA was extracted from PBMCs using a NucleoSpin tissue kit (Macherey-Nagel). The genomic DNA was then amplified by nested PCR using a series of long terminal repeats (LTRs), as well as *gag*, *pol*, *env*, and *bet*/*tas* primers (Table 1). Based on a sequence comparison with SFV from the African green monkey (GenBank accession number MF582544) and guenon (GenBank accession number NC_043445), we ascertained that we had obtained a complete proviral sequence. The SFV strain V909/03F sequence is 13,393 bp long with a GC content of 37.84%. Using PCR products, each nucleotide was sequenced (Eurofins) at least twice on both strands. From the individual PCR product sequences, genome assembly was performed using Genome Compiler software (v2.2.88), NCBI BLASTN, and Clustal W for alignment and analyses. Thirty-five complete SFV sequences available in GenBank were used to make sequence comparisons. The genomic structure organization of V909/03F was similar to that of other SFVs, with the presence of LTRs and *gag*, *pol*, *env*, *tas*, and *bet* sequences. As expected, the genome of strain V909/03F is more closely related to Old World monkey SFV strains, in particular from African animals, than to strains from New World animals (Fig. 1). The Pol amino acid sequence is the most conserved (up to 87% identity) between *P. anubis* and other animal species, while Tas and Bet in the closest strains are only 56% and 63% identical, respectively.

**TABLE 1:** List of primers used to amplify the SFV *Papio anubis* simian foamy proviral sequence

Target gene	Primer	Sequence (5′−3′)
LTR	SFV-TasR4	TTAAAAGGGAGACACTCTGGCTGAGGATGA
	SFV-TasR5	TTGGCACCTCGCCCAAAACCTTAAAGAAGAATA
	SFV-LTR-F1	AAGACTGCACCTTGCATAAAGAGTTC
	SFV-LTR-F2	TTAGATTGTACGGGAGCTCACCA
	FVLGF1-R	CTTACCAAACCTGGAGAGTCTCGAACA
	FVRU5-R	ACTCTCGRCGCAGCGAGYAGTG
	3R	CACGTTGGGCGCCAATTG
*gag*	SFV-GagF1	GGAGGGAAGAACAAAGACCTGTAAATC
	SFV-GagF2	GTTCAAATGCAAAGGGATGAGTTAG
	SFV-GagR3	AGCTCCCCATCCAAAATCGG
	SFV-GagR4	SFV-GagR4 TATCGCTGGAGGAGCACTAGG
	SFV-GagF5	CAAAGGCAATCTGCCCAACCTCAGTC
	SFV-GagF6	GGTAATCAGGGACGTGGTGGATACA
	SFV-GagF	AGGAAGAGGGAACCAAAACCG
*pol*	SFV-PolR8	TGTGTCCTACTTGATTTTCCCAATGTTGCC
	SFV-PolR9	GACTGTACCAGTTGCTATGTGATGAG
	SFV-PolR6	GGATTAAATTGGGTTTCAGCTATAC
	SFV-PolR7	CAGCTGAGCTGTATGATCTCCAAG
	SFVmnd-IntF1	GCCACCCAAGGGAGTTATGTGG
	SFVmnd-IntF2	CCTGGATGCAGAGTTGGATC
	SFVmnd-IntR1	GCTGCACCCTGATCAGAGTG
	SFVmnd-IntR2	GAAGGAGCCTTAGTGGGGTA
*env*	SFV-EnvF1	GTCATAGACTGGAATGT
	SFV-EnvF2	CATCCAGAACCCATAAT
	SFV-EnvF5	CGCAGTTTTCACCCATTGGAT
	SFV-EnvF8	CAATGCCCACTCCCAGGAATACATGAT
	SFV-EnvR6	ATCCAATGGGTGAAAACTGCG
	SFV-EnvF9	TGACTGTCCAGTGACAGCA
	SFV-EnvF10	AGCATATGTACCCAGTGTGGTC
	SFV-EnvR1	CATATGCTGGTATTGAG
	SFV-EnvR3	TTCCCAGGAAGCCATGACA
*bet* and *tas*	SFV-BetR1	GCTAAGATCTGCTAAAGGATTGTCTTCTGGA
	SFV-BetR2	GGTAAGTTTTCTCATTGGAAGGTC
	SFV-BetF3	GATGCTTACACTCGGGGCTACCA
	SFV-BetF4	TTGGCTGTGGCAATGTCAGGA

### Data availability.

The complete genome sequence of SFV V909/03F is available in GenBank under accession number MK241969.
